# Epidemiological profile of Dupuytren’s disease in Taiwan (Ethnic Chinese): a nationwide population-based study

**DOI:** 10.1186/s12891-015-0476-7

**Published:** 2015-02-10

**Authors:** Chin-Choon Yeh, Kuo-Feng Huang, Chung-Han Ho, Kuan-Ting Chen, Cheng Liu, Jhi-Joung Wang, Chin-Chen Chu

**Affiliations:** 1 Division of Plastic Surgery, Department of Surgery, Chi-Mei Medical Center, Yong-Kang, Tainan Taiwan; 2 Department of Medical Research, Chi-Mei Medical Center, Tainan, Taiwan; 3 Department of Hospital and Health Care Administration, Chia-Nan University of Pharmacy and Science, Tainan, Taiwan; 4 Department of Anesthesiology, Chi Mei Medical Center, 901 Zhonghua Road, Yongkang District Tainan, 71004 Taiwan; 5 Department of Recreation and Health-Care Management, Chia-Nan University of Pharmacy and Science, Tainan, Taiwan

**Keywords:** Dupuytren’s disease, Epidemiology, Ethnic, Chinese

## Abstract

**Background:**

The epidemiologic profile of ethnic Chinese patients with Dupuytren’s disease is unknown. We therefore investigated the epidemiology of Dupuytren’s disease using Taiwan’s National Health Insurance Research Database.

**Methods:**

Patients who filed claims for treatment for Dupuytren’s disease between January 2000 and December 2011 were identified in the database. Age- and gender-specific incidences were estimated by dividing the incidence number by population data.

**Results:**

We identified 1,078 patients with Dupuytren’s disease (681 men, 397 women; male/female ratio: 1:1.72). The annual incidence rate ranged from 0.39-0.63/10^5^ for men and 0.14-0.44/10^5^ for women. A trend analysis revealed a rising trend in the annual incidence from 2001 to 2011 (p = 0.0199). The prevalence rate increased steadily from 0.46/10^5^ in 2000 to 4.52/10^5^ in 2011 (p = 0.0186). The mean age at onset was significantly higher in men than in women (60.7 ± 18.4 vs. 53.7 ± 15.5 years). Peak age at onset for men was 70–79 (28.1%) and for women was 50–59 (33.5%). Men > 60 years old had higher incidence rates than did women (incidence rate ratios: 2.0, 4.5, and 6.6 for those 60–69, 70–79, and ≥ 80, respectively). Hypertension (29.6%), diabetes mellitus (21.9%), hyperlipidemia (14.8%), ischemic heart disease (10.5%), and chronic obstructive pulmonary disease (8.0%) were the most common comorbidities.

**Conclusions:**

The incidence and prevalence of Dupuytren’s disease and the male/female ratio were significantly lower in ethnic Chinese than in Western ethnic groups. Moreover, the age at onset was significantly lower in ethnic Chinese women. However, the incidences of three comorbidities (hypertension, diabetes mellitus, and hyperlipidemia) were similar to those in other ethnicities.

## Background

Dupuytren’s disease (DD), also called Dupuytren’s contracture or palmar fibromatosis, is a common and often irreversible and progressive fibroproliferative tissue contraction affecting the palmar fascia [1]. Its typical diagnostic features are the presence of a nodule and the subsequent formation of a contracture cord. The contracture usually begins in the palm and then progresses distally, which results in finger flexion contracture. Nodules can present in both the palm and fingers. The ring finger is most often affected, and then, in order of frequency, the small finger, thumb, middle finger, and index finger [2]. DD is commonly bilateral, and it can become both a physically and a psychosocially disabling condition that significantly affects healthcare resources [3]. The etiology of DD is still not clear. Clinical studies show that it is strongly associated with genetic, geographic, and environmental factors [4]. DD has also been linked to many risk factors, including a history of smoking [5], alcohol consumption [6], frozen shoulder [7], epilepsy [8], diabetes mellitus [9], carpal tunnel syndrome [10], and hand injury [11].

Epidemiological data suggest that the highest prevalence of DD occurs in white northern Europeans. Tradition has it that the disease originated with the Vikings, who spread it throughout northern Europe and beyond as they traveled and intermarried [12]. DD is often called the “Viking” or “Nordic” disease. It is reported to be present in 46% of the population of Norway, 39% of Scotland, 33% of Iceland, and 32% of Finland [13-15]. It is one of the most commonly inherited connective tissue disorders in Norwegians > 60 years old [16]. Nevertheless, the disease does occur in nearly all populations [3,17-24]. The prevalence of Dupuytren's disease is 0.53% (533 per 10^5^) in Hispanics in the USA [23], 28% in Australia [15], 3% in the USA, and > 4% in the male population in England [25]. DD is believed to be rare in Asians. In ethnic Chinese in particular, it is usually described as a sporadic finding, and the epidemiological reports on DD in Asians are limited to small case series reports [19,26]. In a population study in the USA, the prevalence of DD in Asians in one New York City hospital was 0.28% [23]. Given that the prevalence of DD varies greatly based on genetics, geography, and environment [8], the epidemiological data from other countries cannot be applied to ethnic Chinese. Therefore, using a nationwide database in Taiwan, we investigated the epidemiological profiles of DD in ethnic Chinese in Taiwan. Our findings should provide a better understanding of the reported epidemiological trends and comorbid factors of DD in ethnic Chinese.

## Methods

### Database

Population-based data were obtained from the reimbursement claims database of Taiwan’s National Health Insurance (NHI). The NHI program began in Taiwan in 1995 and the NHI database contains annually updated information from 1997 to the present. All medical benefit claims for more than 99% of Taiwan’s 23 million legal residents (citizens and noncitizens) are included. The NHI Research Database (NHIRD) provides encrypted patient identification numbers, gender, date of birth, dates of admission and discharge, ICD-9-CM diagnosis and procedure codes (up to 5 each), details of prescriptions, and costs covered and paid for by the NHI. The NHI Bureau approved our use of these data, and Chi Mei Medical Center hospital institutional review board approval was waived for this study because patient information was encrypted.

### Patients and variables

Patients with DD were identified either from ambulatory visits or from inpatient claims using the ICD-9-CM diagnosis code 728.6. We also recorded respiratory, cardiac, renal, cerebrovascular, and gastrointestinal comorbidities. We counted these as comorbidities if the condition occurred either in inpatient claims or in 2 or more outpatient claims coded 12 months before and after the index medical care date for DD.

Outpatient claim files include an encrypted personal identification number, date of birth, date of service, relevant department, expenses, and the first 3 ICD-9 codes. Hospitalization discharge claims include an encrypted personal identification number, date of birth, length of stay, relevant department, expenses, and the first 5 ICD-9 codes. Multiple outpatient and hospitalization discharge claims with the same encrypted personal identification number were counted once so that only the incidence of the disease was counted. The prevalence was calculated by dividing the number of total cases for each year by the corresponding total population.

Average age- and gender-specific incidence rates were calculated by dividing the number of new cases in each age and gender group by the age- and gender-specific population, and then averaging these data from 2001 to 2011. Detailed age-specific population information for Taiwan used to calculate age-specific incidences in this study is from the Taiwan Ministry of the Interior, Executive Yuan (http://www.moi.gov.tw/stat/english/year.asp).

The communities in Taiwan were stratified into 7 urbanization categories according to the standards published by the Taiwanese National Health Research Institute (NHRI): code 1 = most urbanized and code 7 = least urbanized. These standards include the population density (people/km^2^), the ratio of people with high educational levels (college or above), the ratio of people ≥ 65 years old, the ratio of agricultural workers, and the number of physicians per 100,000 people [27].

### Statistical analysis

SAS 9.3 for Windows (SAS Institute, Cary, NC, USA) was used for this study. Student's *t* test was used to compare continuous variables (age differences between men and women). The incidence rate ratio was estimated using Poisson regression. For categorical variables, a two-tailed Fisher's Exact test was used for < 5 cases (rheumatologic disease, pulmonary tuberculosis, valvular heart disease, and alcoholism) and Pearson's χ^2^ test for > 5 cases (hypertension, diabetes mellitus, hyperlipidemia, ischemic heart disease, chronic obstructive pulmonary disease [COPD], stroke, cancer, hepatitis, and chronic renal failure level of urbanization, and hospital location). The Mantel-Haenszel extension of the χ^2^ test was used for trend analysis. Significance was set at p < 0.05.

## Results

We identified 1078 cases of DD in the NHIRD (103 extant cases in 2000 and 975 new cases during 2001–2011; 681 men and 397 women) (Table 1). The annual incidence rate ranged from 0.39-0.63/10^5^ for men and 0.14-0.44/10^5^ for women. A trend analysis revealed a rising trend in the annual incidence from 2001 to 2011 (p = 0.0199). The incidence rate for men was always higher than for women (average M/F incidence ratio: 1.72 during 2001–2011). The prevalence rate increased steadily from 0.46/10^5^ in 2000 to 4.52/10^5^ in 2011 (p = 0.0186). The prevalence in 2011 was 5.65/10^5^ for men and 3.39/10^5^ for women. Confidence intervals (CIs) are not reported because these are national population data, not a sample.

**Table 1:** **Incidence and prevalence of Dupuytren’s disease in Taiwan by gender**

	Total population × 10^6^		New cases		Total cases		Incidence rate per 10^5^/year		Prevalence per 10^5^		M/F ratio of incidence
Year	M/F	Total	M/F	Total	M/F	Total	M/F	Total	M/F	Total	
2000	11.4*/*10.9	22.3			67/36	103			0.59/0.33	0.46	1.78
2001	11.4*/*11.0	22.4	54*/*15	69	121/51	172	0.47/0.14	0.31	1.06/0.46	0.77	3.47
2002	11.5*/*11.0	22.5	57*/*25	82	178/76	254	0.50/0.23	0.36	1.55/0.69	1.13	2.18
2003	11.5*/*11.1	22.6	47*/*29	76	229/105	329	0.41/0.26	0.34	1.95/0.95	1.46	1.56
2004	11.5*/*11.1	22.7	45*/*35	80	269/140	409	0.39/0.32	0.35	2.34/1.26	1.86	1.24
2005	11.6*/*11.2	22.8	48*/*31	79	314/171	485	0.41/0.28	0.35	2.71/1.53	2.13	1.49
2006	11.6*/*11.3	22.9	49*/*27	76	363/198	561	0.42/0.24	0.33	3.13/1.75	2.45	1.77
2007	11.6*/*11.3	23.0	61*/*23	84	423/220	643	0.53/0.20	0.37	3.65/1.95	2.80	2.58
2008	11.6*/*11.4	23.0	56*/*44	100	475/263	738	0.48/0.39	0.43	4.09/2.31	3.21	1.25
2009	11.6/11.5	23.1	73*/*39	112	545/300	845	0.63/0.34	0.48	4.70/2.61	3.66	1.86
2010	11.6/11.5	23.2	67*/*42	109	607/342	949	0.58/0.37	0.47	5.23/2.97	4.09	1.58
2011	11.6/11.6	23.2	57*/*51	108	655/393	1048	0.49/0.44	0.47	5.65/3.39	4.52	1.12
Total new cases			614*/*361	975							
Total identified cases:			681*/*397	1078							1.72
(new cases from 2001–2011 + extant cases in year 2000)											

M/F, male/female.

The data of total population are from the Directorate General of the Budget, Accounting, and Statistics, Executive Yuan, Taiwan. (http://www.moi.gov.tw/stat/english/year.asp).

Trend analysis 2001–2011: for incidence rate p = 0.0199 and for prevalence rate p = 0.0186 (Mantel-Haenszel extension of the χ^2^ test).

The mean age of patients with DD was significantly higher in men (60.7 ± 18.4 years) than in women (53.7 ± 15.5) (Table 2). The incidence was lowest in the < 40 age group and higher in the older age groups for both genders. The highest incidences were in 70- to 79-year-old men (28.1%) and 50- to 59-year-old women (33.5%). The difference was significantly higher in 60- to 69-, 70- to 79-, and ≥ 80-year-old men than in women in those age groups.

**Table 2:** **Age-specific average incidence of Dupuytren’s disease in Taiwan during 2000–2011**

					Average incidence				
	Cases*n*(%)		Total		per 10^5^/year			M/F incidence	
Age group (years)	M (n = 681)	F (n = 397)	(*n* = 1078)	M/F	M	F	Total	Rate ratio (95% CI)	p
<40	83 (12.2%)	60 (15.1%)	143 (13.3%)	1.38	0.10	0.08	0.09	1.3 (0.9-1.8)	0.1069
40-49	75 (11.0%)	74 (18.6%)	149 (13.8%)	1.01	0.34	0.34	0.34	1.0 (0.7-1.4)	0.9964
50-59	118 (17.3%)	133 (33.5%)	251 (23.3%)	0.89	0.73	0.81	0.77	0.9 (0.7-1.2)	0.3992
60-69	149 (21.9%)	79 (19.9%)	228 (21.2%)	1.89	1.64	0.81	1.21	2.0 (1.5-2.6)	<0.0001
70-79	191 (28.1%)	41 (10.3%)	232 (21.5%)	4.65	2.94	0.65	1.81	4.5 (3.3-6.2)	<0.0001
≥80	65 (9.5%)	10 (12.2%)	75 (2.5%)	6.50	2.37	0.36	1.36	6.6 (3.7-11.7)	<0.0001
Age (mean ± SD)	60.7 ± 18.4	53.7 ± 15.5							<0.0001

M, male; F, female.

Data are *n* (%) unless otherwise indicated.

The most common comorbidities for these 1,078 patients were hypertension (319; 29.6%), diabetes mellitus (236; 21.9%), hyperlipidemia (159; 14.8%), ischemic heart disease (113; 10.5%), and COPD (86; 8.0%) (Table 3). Significantly more men had hypertension (p < 0.001), ischemic heart disease (p = 0.0167), and COPD (p < 0.001) than did women. There were no significant differences between where the hospitals were or the degree of urbanization between men and women.

**Table 3:** **Gender-specific comorbidities, urbanization, and hospital level for 1078 patients with Dupuytren’s disease in Taiwan**

Variables	Total = 1078 (100%)	Male = 681 (100%)	Female = 397 (100%)	*p
Comorbidities (*n* (%))				
Hypertension	319 (29.6)	231 (33.9)	88 (22.2)	<0.0001^$^
Diabetes mellitus	236 (21. 9)	163 (23.9)	73 (18.4)	0.0336^$^
Hyperlipidemia	159 (14.8)	95 (14.0)	64 (16.1)	0.3323^$^
Ischemic heart disease	113 (10.5)	83 (12.2)	30 (7. 6)	0.0167^$^
COPD	86 (8.0)	74 (10.9)	12 (3.0)	<0.0001^$^
Stroke	54 (5.0)	40 (5.9)	14 (3.5)	0.0884^$^
Cancers	37 (3.4)	26 (3.8)	11 (2.8)	0.3624^$^
Hepatitis (B or C)	17 (1.6)	11 (1.6)	6 (1.5)	0.8949^$^
CKD	21 (2.0)	11 (1.6)	10 (2.5)	0.3005^$^
Rheumatic disease	14 (1.3)	3 (0.4)	11 (2.8)	0.0017&
Pulmonary tuberculosis	8 (0.7)	5 (0.7)	3 (0.8)	1.0000&
Valvular heart disease	7 (0.7)	5 (0.7)	2 (0.5)	0.6496&
Alcoholism	1 (0.1)	1 (0.2)	0 (0.0)	1.0000&
Urbanization				0.0980^$^
1 (highest)	275 (25.5)	179 (26.3)	96 (24.2)	
2	567 (52.6)	366 (53.7)	201 (50.6)	
3	38 (3.5)	17 (2.5)	21 (5.3)	
4	171 (15.9)	101 (14.8)	70 (17.6)	
≥5	27 (2.5)	18 (2.6)	9 (2.3)	
Hospital location				0.4407^$^
1. North	579 (53.7)	375 (55.1)	204 (51.4)	
2. Central	158 (14.7)	95 (14.0)	63 (15.9)	
3. South	316 (29.3)	193 (28.34)	123 (31.0)	
4. East	25 (2.3)	18 (2.6)	7 (1.8)	

COPD: chronic obstructive pulmonary disease. CKD, chronic kidney disease.

Urbanization: level 1 indicating most urbanized and level 7 indicating least urbanized. Criteria for calculating urbanization include population density (people/km^2^), ratio of people with high educational levels (college or above), the ratio of people ≥ 65 years old, the ratio of agricultural workers, and the number of physicians per 100,000 people.

*Men compared with women; &Fisher’s Exact test; ^$^Pearson's χ^2^ test.

## Discussion

This is not only the first population-based epidemiological study on DD in Taiwan, but it is also is the largest epidemiological study that has ever been conducted on DD in an Asian country, and one of very few that uses as its source one nation’s entire stock of national health insurance claims data. Because of the low incidence, it is difficult and impractical to field community studies for an epidemiological approach. The NHIRD, however, is a prodigious resource for studying the epidemiology of rare diseases like DD. We found that both the incidence and prevalence rates of DD are much lower for ethnic Chinese in Taiwan than for Europeans [14,28,29] and US Americans [24,30]. However, the annual incidence rate of DD in Taiwan increased during the study period, especially for women. We also found that the most common comorbidities in ethnic Chinese DD patients were hypertension, diabetes mellitus, hyperlipidemia, ischemic heart disease, and COPD.

Other studies’ estimated prevalence rates of DD ranged from 0.2% [31] to 56% [32]; the large range is attributable to variations in age, population groups, and methods of data collection. Most of the epidemiological data were obtained by sampling specific populations [14,21,24,33] or by retrospectively reviewing charts [23,34]. For instance, one US study [24] estimated the prevalence rate of DD using an online survey from an Internet-based research panel representative of the US population, and one Belgian study [14] estimated it from a random visit by a physician to a market. Our estimate, however, is based not on a sampling, but on data from Taiwan’s NHIRD, into which data are entered without a selection bias. No similar information is available in the published literature in Taiwan or in any other Asian country. Because the diagnosis of DD was already recorded using ICD-9 codes, and because each diagnosis was made by a physician rather than being based on the patient’s memory on a health history questionnaire, a misclassification bias is unlikely. Because patients with only mild symptoms might not seek medical help, and therefore will not be entered in the database, the incidence and prevalence may be underestimated. However, DD is a proliferative and progressive disease, and most people who have it will ultimately need medical attention. Therefore, if the observation period is long enough, the underestimation will become smaller. Furthermore, because more than 98% of Taiwan’s population is ethnic Chinese [35], our data can represent the epidemiological features of DD in ethnic Chinese.

The two main foci of epidemiological studies are incidence rate and prevalence rate. The former is defined as the number of new cases of a disease over a specific time-period and the latter as the current number of cases of disease at a single point in time. Most previous epidemiological studies of DD that have quoted incidence rates were in fact referring to prevalence rate [15]. In the present study, we identified new cases every year from the NHIRD. The average annual incidence rate of DD in Taiwan during 2000–2011 was only 0.39/10^5^ (men 0.48/10^5^; women 0.29/10^5^), which was much lower than that of a UK study [29] (502,493 men; annual incidence: 34.3/10^5^ men), and of a US population-based study [24] (annual incidence: estimated at 30.0/10^5^ adults). We found that DD in Taiwan was much less prevalent than in Western countries [14,21,22,33]. Ethnic studies [14,17,21] show that DD is highly prevalent in Scandinavians. A US veterans-hospital-based study [30] reported that the prevalence of DD is highest in Whites and then Hispanic Whites, Blacks, and Asians.

The epidemiological reports on DD in Asia are only case series or else are limited to special groups. A Taiwanese study [19] reported 41 cases of DD in Taiwan during 1970–1988. Because it was case series from a single hospital, neither an incidence rate nor a prevalence rate could be reported. In addition, a Japanese cross-sectional study [36] of 1,154 patients reported prevalence rates of 19.7% for men and 9% for women. Because all the patients were more than 60 years old and lived in nursing homes, they did not represent a general population.

In the present study, we found a trend of rising incidence and prevalence rates from 2001 to 2011. We hypothesize that the trend is a reflection of the aging population because the prevalence and severity of DD increases with age [37]. Moreover, the incidence of DD increases with comorbidities like diabetes mellitus [9,31], hyperlipidemia [38,39], and hypertension [23,40], all of which are highly prevalent in the elderly [41-44]. Furthermore, the universal coverage and low copayment of Taiwan’s NHI system has increased the affordability of healthcare here, which increases the diagnostic rate for DD. In addition, increased medical knowledge from the Internet might also contribute to the higher contemporary rate of medical consultation [45].

The average age at the onset of DD is about 60 years old, and the incidence rises thereafter [13,37]. Similarly, in our study, the onset of DD age-dependently increased for men, and the incidence rate peaked at 2.94/10^5^ person-years at age 70–79 and dropped a little to 2.37/10^5^ person-years for men over 80. For women < 40 years old, however, the incidence rate jumped from 0.08/10^5^ to 0.81/10^5^ person-years for those 50–69 years old, and fell by 20% to 0.65/10^5^ person-years for those 70–79 and then by 55% to 0.36/10^5^ person-years for ≥ 80. These rates are different from those reported [46,47] for other ethnicities, in which the incidence continuously increased with age in women.

Another unique epidemiology profile for DD in ethnic Chinese is that there are no gender differences in incidence rates for patients < 60, but for those ≥ 60, the incidence rates increased more dramatically for men than for women. The male:female incidence rate ratio increases from 2.0 to 6.6 for those 60–69 and ≥ 80 years old. This is quite different from reports on other ethnicities in which the gender difference in the incidence rate diminishes with increasing age; specifically; the male:female incidence rate ratio approaches 1:1 for people ≥ 80 [17,25,46,47]. Additional studies are necessary to determine the underlying mechanisms.

Generally, DD is much less common in women than in men, with reported overall male:female incidence rate ratios ranging from 1.7:1 to 9.5:1[37,46,47]. Similar to what has been reported in the literature, our results show that both the incidence and prevalence rates are higher for men than for women in Taiwan. The male:female incidence and prevalence rate ratios, however, are 1.72 and 1.55, which are lower than those for Europeans and Australians [37,46], but close to that for US Americans [47]. Additionally, a US study [46] reported that women were older (mean age: 62.4 years) at their first operation (62.4 years) than were men (59.8 years). Other studies [14,21] also report that age at the onset of DD is about 10 years lower in men than in women. However, in the present study, the mean age at onset was about 7 years lower in women (53.72 years) than in men (60.69 years). This, however, might be attributable to genetic and environmental differences between populations. Moreover, the present study is an epidemiological profile report of DD in Taiwanese; thus, from our results, no direct evidence can be drawn determine the underlying mechanism for the early onset of DD in Taiwanese women.

A number of epidemiologic factors have been associated with the development of DD: e.g., diabetes mellitus, hyperlipidemia, hypertension, epilepsy, smoking, and alcoholism [13,15,37,48]. We found that hypertension, diabetes mellitus, and hyperlipidemia were the most common comorbidities both in men and in women. All of these diseases may impair microvascular circulation and cause localized ischemia, which increases free radicals and inflammatory cytokines [37], and concomitantly alters gene expressions of growth factors, adhesion molecules, and extracellular matrix components that, in turn, trigger the proliferation of DD tissue [49].

Our study has some limitations. First, our cohort was identified by ICD-9 codes coded by physicians. Patients with only mild symptoms might not seek medical care and thus were not included in our database. Therefore, the calculation of incidence and prevalence rates based on the NHIRD might underestimate the true rates. Second, data on several environmental factors believed to contribute to DD development, including alcohol intake, smoking, manual labor, or exposure to vibrations [37], were not available in the NHIRD. Only one man among our identified 1,078 patients had been diagnosed with alcoholism; this is undoubtedly an underestimated prevalence rate of habitual alcohol drinking for Taiwan. Third, although the administrative claims data allowed us to identify patients diagnosed with DD, they did not contain information about the severity of the disease. Fourth, there were no data about blood biochemistry, pathology reports, or finger function; thus, it was not possible to determine whether the disease was progressing or in remission. Fifth, although 98% of Taiwanese residents are ethnic Chinese, there are residents of other ethnicities, most of whom are aboriginal Austronesian Taiwanese [35]. Patients’ ethnicities were not recorded in the NHIRD; therefore, we did not know the distribution of DD in ethnic Chinese and aborigines. However, the highest population density of Taiwanese aborigines is in eastern Taiwan, and DD is lowest in that area. Sixth, although the NHI regularly investigates the quality of the ICD-9 coding in the claims database to ensure that diagnoses in particular are accurate, we still cannot exclude the possibility that some might be incorrect because of mistakes by the medical coders and physicians responsible for entering the codes. These limitations may compromise our findings.

## Conclusions

The incidence and prevalence rates of DD in ethnic Chinese in Taiwan were much lower than those reported for other ethnic groups in the rest of the world. Moreover, DD in Taiwan has an earlier onset in ethnic Chinese women and a lower M:F incidence rate ratio. However, diabetes mellitus, hyperlipidemia, and hypertension, the most common comorbidities of DD in ethnic Chinese, were similar to those in other ethnic groups. Additional studies are needed in order to determine, for example, whether the underlying mechanisms are genetic, environmental, or socioeconomic factors, or some combination of these.
